# Similar or Different? The Role of the Ventrolateral Prefrontal Cortex in Similarity Detection

**DOI:** 10.1371/journal.pone.0034164

**Published:** 2012-03-30

**Authors:** Béatrice Garcin, Emmanuelle Volle, Bruno Dubois, Richard Levy

**Affiliations:** 1 CR-ICM – UPMC, Inserm UMR_S 975;CNRS UMR 7225, Groupe Hospitalier Pitié-Salpêtrière, Paris, France; 2 AP-HP Service de Neurologie, Hôpital Pitié-Salpêtrière, Paris, France; 3 AP-HP Service de Neurologie, Hôpital Saint-Antoine, Paris, France; 4 Centre de NeuroImagerie de Recherche - CENIR, CR-ICM - UPMC UMR-S975, Inserm U975, CNRS UMR7225, Groupe Hospitalier Pitié-Salpêtrière, Paris, France; French National Centre for Scientific Research, France

## Abstract

Patients with frontal lobe syndrome can exhibit two types of abnormal behaviour when asked to place a banana and an orange in a single category: some patients categorize them at a concrete level (e.g., “both have peel”), while others continue to look for differences between these objects (e.g., “one is yellow, the other is orange”). These observations raise the question of whether *abstraction* and *similarity detection* are distinct processes involved in abstract categorization, and that depend on separate areas of the prefrontal cortex (PFC). We designed an original experimental paradigm for a functional magnetic resonance imaging (fMRI) study involving healthy subjects, confirming the existence of two distinct processes relying on different prefrontal areas, and thus explaining the behavioural dissociation in frontal lesion patients. We showed that: 1) *Similarity detection* involves the anterior ventrolateral PFC bilaterally with a right-left asymmetry: the right anterior ventrolateral PFC is only engaged in detecting physical similarities; 2) *Abstraction per se* activates the left dorsolateral PFC.

## Introduction

Categorization is essential to organize semantic content in a meaningful way for everyday perception, action and decision-making. Human categorization has been widely studied during the past 15 years, and the current theories hold that humans have multiple category learning systems including rule-based and similarity-based categorization [1], [2]. Here, we address the question of categorization from a totally different view, based on the clinical observation of patients with frontal lobe lesions.

When asked “In what way are an orange and a banana alike?”, patients with frontal lobe lesions frequently provide two types of abnormal answers (see Videos S1). Some patients do not find any similarities and keep looking for differences between the items: “they are not alike: one is yellow, the other is orange” or “their shapes are different” [3]. In other words, they are stuck in a discrimination processing mode, and are no longer capable of *similarity detection*, defined as a process (or a set of processes) by which different objects are perceived as sharing one or several common (physical or abstract) features. Other patients do detect similarities but only at a concrete level: “both have peel” or “they are sweet” [3]. Although one can consider theses answers appropriate, they differ from that of normal controls who point out to abstract similarities (the taxonomic category of the two objects, in the present example). The frontal patients behave as if they are unable to access the abstract level that characterizes these items (e.g. “both are fruits”). This suggests a deficit in *abstraction* - a process (or a set of processes) that allows objects to be placed within a conceptual class that surpasses their physical features.

Although *similarity detection* and *abstraction* are both required to classify items within abstract categories, they are different and sometimes independent processes. Indeed, it is possible to detect similarities without abstraction (for instance, if one is asked what objects are the most similar in shape). The reverse is also true: it is possible to use abstract thinking without looking for similarities (for instance, if one is asked what objects do not belong to a given abstract category). Similarity detection *per se* has not been studied earlier as a cognitive function, and the involvement of cognitive control and executive functions for similarity detection is undetermined. No assumption was made about the PFC regions involved in similarity detection. Abstraction is necessary for complex goal-directed behaviour and can be considered as part of the executive functions, which is known to involve the lateral prefrontal cortex. Functional imaging studies in healthy humans have shown the involvement of the lateral prefrontal cortex (LPFC) in abstract categorization. In these studies, subjects were asked to identify abstract or conceptual relationships between stimuli, and the processes involved in *abstraction* and *similarity detection* were intermingled [4]–[10]. To our knowledge, there has been no attempt as yet to distinguish between *abstraction* and s*imilarity detection*. The fact that some patients with PFC lesions cannot find similarities while others cannot come up with abstract concepts raises the question of whether there are two different anatomical/functional prefrontal modules involved in categorization: one devoted to *similarity detection* and the other involved in generating or providing access to concepts (*abstraction*). If so, it is also of importance to precise the nature of the interaction between these different anatomical/functional prefrontal modules for abstract categorization. To answer these questions, we performed a functional MRI (fMRI) study with an experimental paradigm designed to distinguish *abstraction* from *similarity detection*.

## Materials and Methods

### 1. Subjects

Twenty healthy individuals (aged 20 to 33 years, 10 women and 10 men, right-handed with normal or corrected-to-normal visual acuity) participated in the study. All subjects were native French speakers and all subjects had studied at least 2 years at university. Subjects were excluded if they had been diagnosed with a past or present psychiatric or neurological disorder. The study was approved by the ethical committee “Comité de Protection des Personnes d'Ile de France VI”, and each subject gave written informed consent.

### 2. Experimental tasks

**Figure 1:**
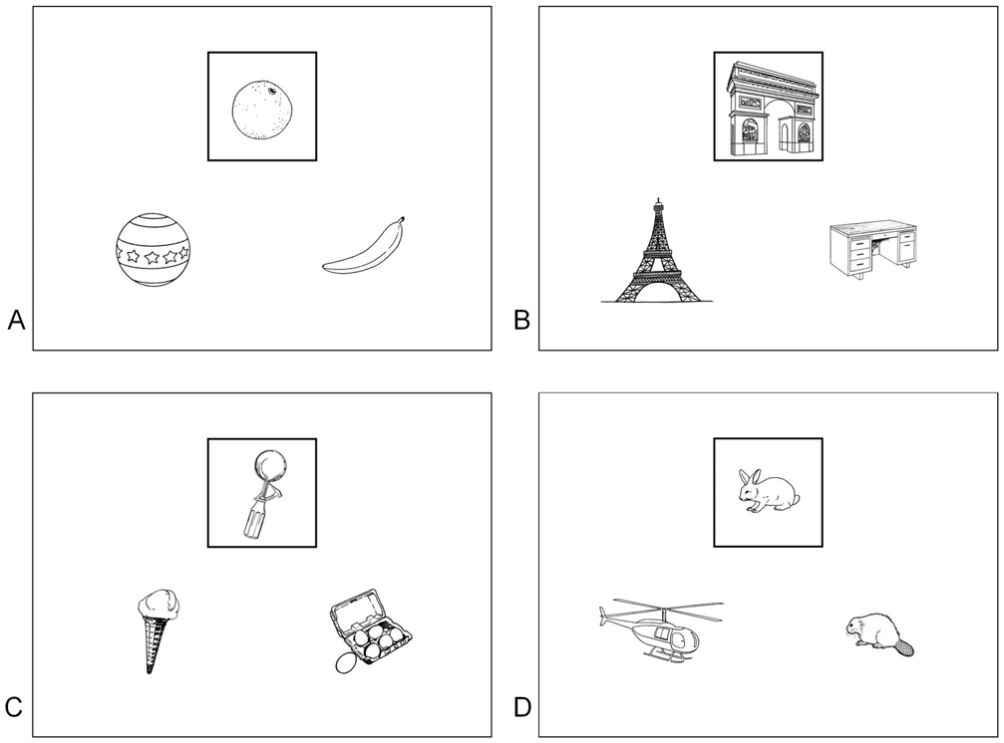
Samples of stimuli. The framed drawing was compared with the two bottom ones. There was systematically an abstract and/or a shape relationship between the framed drawing and at least one of the two others. In half of the stimuli, one drawing had a similar shape, whereas the other one belonged to the same category as the framed drawing (“non matching slides”), such as in stimuli A and B. In the other half, the drawing with the most similar shape belonged to the same category as the framed one (“matching slides”), such as in stimuli C and D. Some categories were taxonomic such as in stimuli A (“fruits”), B (“monuments”) and D (“rodents”), while others were thematic, such as in stimulus C (“functional link”). Among all drawings, two thirds were non-living objects and one third were living objects. Some objects were easy to handle (e.g., tools, fruit…) such as in stimuli A and C, while others were not (e.g. buildings, wild animals…), such as in stimuli B and D.

We designed a paradigm which allowed us to separately assess the processes of S*imilarity detection* and *Abstraction*. The paradigm consisted of the presentation of 576 visual stimuli followed by the recording of behavioural responses during an fMRI session. Each stimulus and the subsequent behavioural response represented a “trial”. The stimuli were slides containing three black-and-white drawings of real-life objects. Two of these drawings were located at the bottom of the screen, on the left and right side, respectively. The third was centrally located at the top of the screen and was framed. Participants had to compare the framed drawing with the two other drawings and provide a behavioural response that depended on the task condition. Four experimental conditions were used. For the *same shape* condition, participants had to answer the following question “Which element has the most similar shape to that of the framed drawing?” For the *same category* condition, participants had to answer the following question: “Which element belongs to the same category as the framed drawing?” For the *different shape* condition, participants had to answer the following question: “Which element has the most different shape from that of the framed drawing?” In the *different category* condition, participants had to answer the following question: “Which element does not belong to the same category as the framed drawing?” For each trial, there was a semantic link between the framed drawing and one of the two bottom ones, as well as a similarity of shape between the framed drawing and one of the two bottom ones (for more information, see the legend of Fig. 1 and Text S1). There were 240 different categories. Some categories were taxonomic (e.g. fruits or insects), while others were thematic (e.g. rugby or transportation). The drawings were chosen from among hundreds, and for each trial, the combination of drawings varied (Fig. 1). Participants were provided with an answer button in each hand and were instructed to press a button with their thumb according to the answer: the left–hand button for the bottom-left drawing and the right-hand button for the bottom-right one. In order to balance motor activation between the left and right sides, an equal number of correct responses were located at the bottom-left and bottom-right for every condition (see Text S2 and Table S1).

The fMRI session consisted of 64 blocks, each made up of 9 trials for a given condition. On the whole, 576 trials were performed (144 for each condition). The 576 stimuli were presented in a random order. To avoid any bias due to the repetition of stimuli, a given stimulus was used under only one condition for each participant, and was distributed throughout the four conditions across the population of participants. In addition, for each participant, the order of each block was randomized. Each block started with the presentation of an instruction cue (5000 msec), indicating to the subject the condition of the 9 subsequent trials (e.g. “*same shape*”). The duration of each trial (presentation and response) was 3500 msec. Participants were required to provide their response during this time interval. A blank screen of 5000 msec was presented between blocks. The experimental paradigm followed a factorial design crossing “*similarity detection*” and “*abstraction*”. The two “*same*” conditions (*same shape* and *same category*) explored *similarity detection* according to the concrete (shape) or the abstract (category) dimension linking the framed drawing with one of the two others. The two “*category*” conditions (*same category* and *different category*) explored *abstraction*.

### 3. Behavioural data acquisition

Stimulus presentation was programmed on a PC using meyeParadigm 1.5 software (e(ye)BRAIN, Ivry-sur-Seine, France, www.eye-brain.com). Stimuli were projected from an EMP-8300 video projector (Epson, Nagano, Japan) outside the MRI room onto a translucent screen located at the end of the scanner bore. Subjects could view the screen with a total path length of 60 cm through a mirror attached to the head coil. The answer buttons were connected to the PC and the meyeParadigm software recorded reaction times (RTs) and accuracy. RTs were measured from the moment the target was presented until the participant made a motor response. Data (RTs and accuracy) were statistically analyzed using repeated measures ANOVA with “task condition” as an independent variable. Tukey's post hoc analyses were performed for comparisons between conditions. Paired t-tests were used for comparisons between *Category* and *Shape* or *Same* and *Different* stimuli. All statistical tests were performed with GraphPad Prism software (GraphPad software, www.graphpad.com), with a threshold of significance of p<.05 two-tailed.

It should be noted that prior to the experiment, subjects underwent a 20-minute training session using specific stimuli that were not used in the experiment.

### 4. Image acquisition and analysis

#### 4.1. Image acquisition

T2*-weighted echo planar images (EPI) were acquired with blood oxygen level-dependant (BOLD) contrast on a 12-channel 3 Tesla scanner (Siemens Trio). For each participant, a total of 1280 EPI-scans were acquired, lasting about 45 minutes. The scanning was divided into 8 runs, each containing 8 blocks. The field of view was parallel to the AC/PC line. To cover the whole brain with a repetition time of 2140 msec, we used the following parameters: 37 slices; 2 mm slice thickness; 1 mm inter-slice gap. T1-weighted structural images were also acquired, co-registered with the mean EPI, segmented and normalized to a standard T1 Montreal Neurological Institute (MNI) template; and averaged across all subjects to allow group-level anatomical localization. EPI images were analyzed in a block manner, within a general linear model, using the Statistical Parametric Mapping (SPM) software SPM5 (Wellcome Department of Imaging Neuroscience, www.fil.ion.ucl.ac.uk/spm) [11]. Pre-processing consisted of spatial realignment, normalization with the same transformation as structural images, and spatial smoothing using a Gaussian kernel with a full-width at half-maximum of 8 mm. Functional images were corrected for slice acquisition time and for head movements. High-pass filters (cut-off period of 384 sec) were applied to reduce the effect of slow signal drifts. For each experiment, statistical analyses at the first level were calculated using a block-related design, with 4 types of blocks (*same shape*, *different shape*, *same category*, *different category*) and eight runs. Blocks were modelled using a canonical hemodynamic response function (HRF). The model also included six covariates per run to capture residual movement-related artifacts. Contrasts of regression coefficients were computed at the individual subject level and then used for a group-level random effect analysis. Contrasts between tasks were evaluated with t-tests and then converted into z-scores.

#### 4.2. Whole-brain analysis

to uncover the neural network involved in *abstraction*, we contrasted tasks involving “abstract judgment” (*same or different category)* to those relative to “shape analysis” (*same or different Shape*). To reveal the neural network involved in *similarity detection*, we contrasted tasks in which participants were asked to indicate similarities between drawings (s*ame shape or category*) to tasks in which participants were asked to indicate differences (*different shape or category*). Reverse contrasts (*same and different shape vs. same and different category* and *different shape and category vs. same and different shape*) were also carried out in order to evidence the neural networks involved in *shape analysis* and *difference detection* respectively, on the assumption that the two latter processes activated different neural networks than those involved in *abstraction* and s*imilarity detection*. Functional activation at the group level was localized with the software MRIcron (www.sph.sc.edu/comd/rorden/mricron/) and the SPM5 toolbox Anatomy (www.fz-juelich.de). All contrasts exceeded an uncorrected threshold of *p*<.001. Clusters were considered significant with a {t} threshold of 3.58, and a “k” extent of 150 voxels. As the “k” extent threshold was estimated using resels, all clusters reached significance after correction for multiple comparisons (p<.05). Interactions between the *shape/category* and *same/different* dimensions were initially evaluated using sample t-tests based on the following contrasts: (*same category* – *different category*) - (*same shape* – *different shape*) and (*same shape* – *different shape*)-(*same category* – *different category*).

#### 4.3. Region of interest (ROI) analyses

Further analyses were performed in order to determine whether the regions highlighted by the contrast *similarities>differences* also participated in *abstraction or shape analysis*, and whether the regions evidenced by the contrast *category>shape* also participated in *similarity or difference detection*. For this purpose, we selected ROIs in the following regions: the two ventrolateral prefrontal clusters identified by the *same*>*different* contrast (right and left anterior VLPFC) and the two closest clusters identified by the *category*>*shape* contrast (left posterior VLPFC and left DLPFC). ROIs were spheres of 8 mm radius defined by the maxima of each cluster of activation in each of the selected regions. Parameter estimates were extracted separately for each subject using the MarsBaR toolbox (http://marsbar.sourceforge.net) [12]. We then performed two-way ANOVAs on the parameter estimates extracted from each ROI, orthogonally crossing *category (different and same)/shape (different and same)* and *same (shape and category)/different (shape and category)* dimensions.

## Results

### 1. Similarity detection

**Figure 2:**
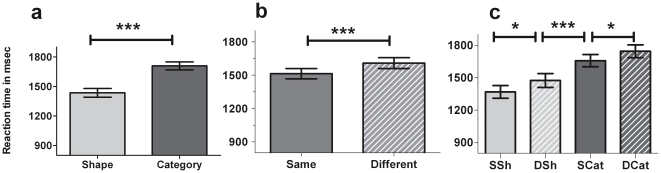
Reaction times (fMRI study). Histograms represent means ± standard errors of the mean. *:*p*<.05; **:*p*<.01; ***:p<.001. *SSh*: Same Shape; *DSh*: Different Shape, *SCat*: Same Category; *DCat*: Different Category. a. Reaction times for *shape* (*SSh* and *DSh*) and *category* (*SCat* and *DCat*) conditions. Paired *t*-tests were used for comparisons. b. Reaction times for *same* (*SSh* and *SCat*) and *different* (*DSh* and *DCat*) conditions. Paired *t*-tests were used for comparisons. c. Reaction times under the four different conditions. ANOVAs were performed for comparisons. Tukey's post hoc analysis confirmed a difference between conditions.

Mean errors were at very low levels and are reported in figure S1. Mean reaction times (RTs) (+/− standard deviation) were shorter for *same* (*shape*+*category*) than for *different* (*shape*+*category*) conditions, with a mean difference of 96 msec (t [19] = 7; *p*<.0001; Fig. 2b). The best performance (i.e. quickest response) was observed in the *same shape* condition, followed by *different shape*, *same category* and *different category* conditions (F [3,19] = 55.4; *p*<.0001; Fig. 2c). ANOVA with repeated measures did not reveal significant differences in RTs across the 8 sessions of the experiment (Figure S2).

**Figure 3:**
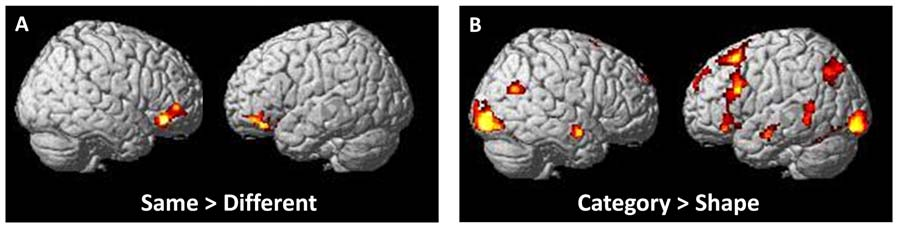
Activation during *abstraction* and *similarity detection*. Activation is displayed on a rendered brain. Only clusters surviving a family-wise error (FWE) correction (p<.05) are reported (cluster extent: 150 voxels). Details regarding activated foci are displayed in table 1.

In whole brain analyses, *same* (*shape*+*category*) and *different* (*shape*+*category*) conditions were contrasted to examine the networks involved in *similarity detection per se*. Significant activation was seen bilaterally in the anterior Ventrolateral Prefrontal Cortex (VLPFC), when performing the contrast *same>different*. More specifically, the left and right orbital frontal cortices (BA 11/47) and the right inferior frontal gyrus (BA 45/46) were seen to be activated (Fig. 3a).

With the opposite contrast (*different*>*same*), no prefrontal activation above the threshold of significance was detected, but activation was significant in the right superior parietal lobule and the precuneus.

### 2. Abstraction

RTs were longer for *category* (*different*+*same*) than for *shape* (*different*+*same*), with a mean difference of 280 msec (t [19] = 8.62; *p*<.0001; Fig. 2a). In whole brain analyses, the contrast between *category* (*same*+*different*) and *shape* (*same*+*different*) conditions was examined in order to reveal networks involved in *abstraction per se*. Activated areas included several large clusters in the left dorsolateral (BA 8/9/10) and left mid VLPFC (BA 44/45/47, a different and more caudal area than that activated by similarity detection *per se*). Bilateral activation was also seen in the fusiform gyri (BA19/21/22), angular gyri (BA 39), medial temporal lobes (BA 21, 22, 39) and occipital lobes (BA 18/19) (Table 1 and Fig. 3b). As RTs were found to be higher in the *category* conditions, we wanted to verify whether the longer RTs could have driven activation. To do this, the same contrast was performed using RT as a variable of non-interest. Prefrontal activation was not much affected (Table S2).

**Table 1:** Results of the main contrasts of interest.

*Contrast*	*Region*	*Side*	*BA*	*MNI coordinate*	*z*
**Category>Shape**					
	middle/superior frontal gyrus	L	8/9	−28 22 52	4.78***
	inferior frontal gyrus	L	44/45/47	−52 22 38 −42 32 −14	4.44***
	superior frontal gyrus	L	9/10	−4 60 38	4.21*
	supplementary motor area	L	6	0 14 62	4.29***
	middle temporal gyrus/fusiform gyrus/cerebellum	R	21/22	56 −4 −14	4.76***
	fusiform gyrus/cerebellum	L	37/19	−32 −48 −20	3.59***
	fusiform gyrus/cingulate gyrus	L	37/30	−26 −36 −20	4.39***
	angular gyrus/middle occipital gyrus	L	7/39	−36 −66 40	4.41***
	middle temporal gyrus	L	21	−54 −44 −4	4.15**
	middle temporal gyrus/angular gyrus	R	39	42 −58 24	4.07**
	inferior/middle occipital gyrus	R	18/19	34 −94 −10	4.71***
	inferior/middle occipital gyrus	L	18/19	−28 −98 12	4.51***
**Shape>Category**					
	supramarginal gyrus/inferior parietal lobule	L	2/40	−60 −30 42	5.36***
	supramarginal gyrus/inferior parietal lobule	R	2/40	56 −24 38	5.90***
**Same>Different**					
	inferior frontal orbital cortex/inferior frontal gyrus	R	45/46/47	48 46 0	4.60***
	inferior frontal orbital cortex	L	47	−44 46 −12	4.32**
	inferior frontal orbital cortex	L	11	−22 20 −12	4.05*
**Different>Same**					
	superior parietal lobule/precuneus	R	5/7	12 −62 62	4.04***

The table shows all clusters surviving a FWE correction (*p*<.05).

* : *p*<.05;

** : *p*<.01;

*** : *p*<.001.

With the opposite contrast (*shape*>*category*), no prefrontal activation above threshold was detected, while bilateral activation was detected in the supramarginal gyrus and the inferior parietal lobule (Table 1).

### 3. Interactions between similarity detection and abstraction

**Figure 4:**
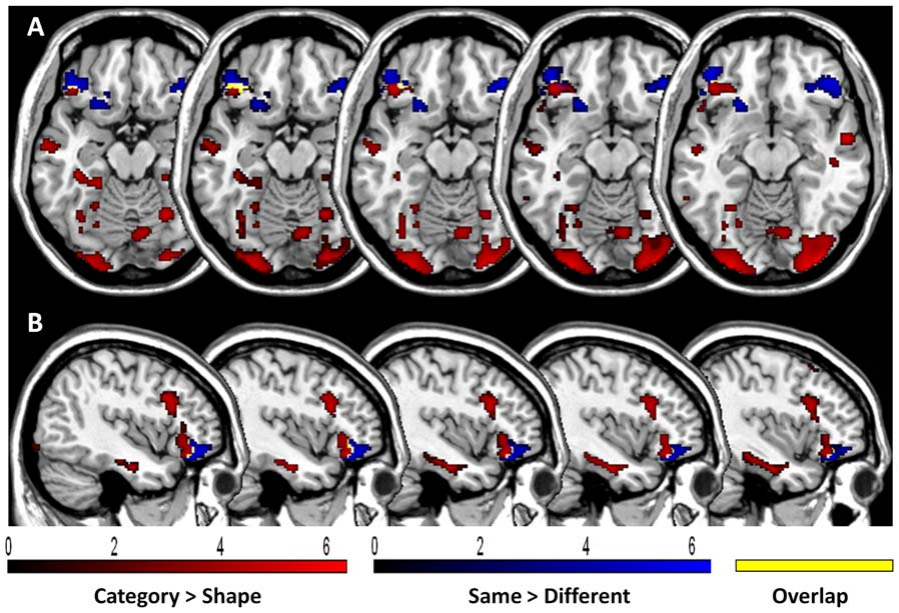
Superimposition of activated areas during *Abstraction* and *Similarity detection*. Coronal (A) and sagittal slices (B) display brain regions activated by *abstraction* in red and brain regions activated by *similarity detection* in blue. The overlap between areas activated during *abstraction* and *similarity detection* is represented in yellow. Only clusters surviving a FWE comparison (p<.05) are reported (cluster extent: 150 voxels).

The results of the whole brain analyses above suggest that *similarity detection* and *abstraction* rely on different brain networks. However, a few areas of overlap between the two types of processes were seen, mainly in the left and posterior prefrontal cortex (Fig. 4). In order to verify whether *similarity detection* and *abstraction* engaged different processes and prefrontal regions, we then analyzed the interaction between *shape/category* and *same/different* effects. This analysis did not reveal any significant interaction.

### 4. Regions of interest (ROIs)

**Figure 5:**
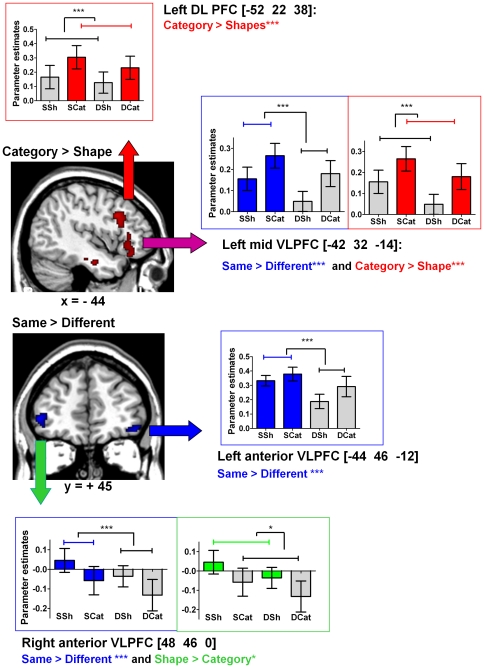
Analysis of variance in the Regions of Interest (ROIs) in the Ventrolateral PFC. Analyses of variance (ANOVAs) were performed for the ventrolateral regions of interest (ROIs) to examine their activation profile during *Similarity detection* and *Abstraction*. *: *p*<.05; **:*p*<.01; ***:*p*<.001. *SSh*: Same Shape, *DSh*: Different Shape, *SCat*: Same Category, *DCat*: Different Category. DLPFC: dorsolateral prefrontal Cortex; VLPFC: Ventrolateral Prefrontal Cortex. In each ROI, two-way ANOVAs were performed to compare activation across the conditions. *Shape/Category* effect: There was significantly more activation in the left DLPFC (*p*<.001) and left posterior VLPFC during *category* than during *shape* tasks (*p*<.001). There was a significantly greater signal change in the right anterior VLPFC during *shape* than during *category* tasks (*p* = .025). There was no *shape/category* effect on activation in the left anterior VLPFC. *Same/Different* effect: There was significantly more activation during *same* than during *differen*t tasks in the left posterior VLPFC (*p*<.001), left anterior VLPFC (*p* = .0000), and right anterior VLPFC (*p*<.001). Interactions: There was no interaction between *similarity detection* and *abstraction* in the ROIs selected.

Further analyses were performed in order to determine: 1) whether or not the left and right anterior VLPFC, involved in *similarity detection*, also participated in *abstraction*, and 2) whether or not the left mid-VLPFC and the left DLPFC, involved in *abstraction*, also participated in *similarity detection*. For this purpose, we selected regions of interest (ROIs) in the following areas: the two largest activated prefrontal clusters identified by the *same*>*different* contrast (right and left anterior VLPFC) and the two most ventral clusters identified by the *category*>*shape* contrast (left mid VLPFC and left DLPFC) (Fig. 5). We then performed two-way ANOVAs on the parameter estimates extracted for each ROI, orthogonally crossing *category (different and same conditions)/shape (different and same conditions)* and *same (shape and category conditions)/different (shape and category conditions)* dimensions.

These analyses did not reveal any interaction between the two dimensions in the four ROIs selected. There was a *same/different* effect, with significantly higher activation in *same* conditions in ROIs identified by the *same* versus *different* contrast, i.e. the right (F[1,19] = 27.11, *p*<.0001) and left anterior VLPFC: (F[1,19] = 20.40, *p*<.0001) (Fig. 5). In ROIs identified by the *category* versus *shape* contrast, i.e. the left mid-VLPFC and the left DLPFC, we observed a *category/shape* effect (F[1,19] = 16.6, *p* = .0006 and F[1,19] = 24.3, *p*<.0001 respectively), with these two ROIs showing greater activation under *category* conditions (Fig. 5). Together, these results further support those of the whole brain analyses showing that two different sets of prefrontal regions are associated with *similarity detection* (left and right anterior VLPFC) and *abstraction* (left mid-VLPFC and left DLPFC).

A *category/shape* effect was also observed in the right anterior VLPFC with more activation in *shape* than in *category* conditions (F[1,19] = 5.87, *p* = .025) (Fig. 5). The right anterior VLPFC was more activated by the *same shape* condition, where subjects were asked to find similarities based on physical features (i.e. shape) (Fig. 5). The left anterior VLPFC was equally and significantly activated by the two *same* conditions (*shape* and *category*) (Fig. 5). These findings indicate a left/right asymmetry depending on the nature of the *similarity detection* performed (*shape* or *category*). Finally, differences in activation were observed between ROIs involved in *abstraction*: in the left mid-VLPFC, but not in the left DLPFC, there was a *same/different* effect (F[1,19] = 16.6; *p* = .0006). As depicted in Fig. 5, in the left mid-VLPFC, activation was higher in both *category* versus *shape* and *same* versus *different* tasks, while in the left DLPFC, the difference between *same* and *different* tasks did not reach significance. These findings show that the left mid-VLPFC is involved in both *abstraction* and *similarity detection* while the left DLPFC is involved only in *abstraction*.

### 5. Summary of the main results

As a whole, the findings of this study show: 1) left and right anterior VLPFC activation associated with *similarity detection (same/different effect)* with no *category/shape* effect; 2) a left/right asymmetry, with the right anterior VLPFC being more activated by shape and the left anterior VLPFC being activated by both shape and category similarities; 3) a left DLPFC activation in tasks involving *abstraction* (*category/shape* effect) with no *same/different* effect; 4) a left mid-VLPFC activation in tasks involving *abstraction* or *similarity detection (same/different effect)* (Fig. 5). This area is anatomically in an intermediary location between the left anterior VLPFC associated with *similarity detection* and the left DLPFC associated with *abstraction*.

## Discussion

By disentangling *similarity detection* and *abstraction* during categorization tasks, we show that the two processes are partially dissociated both functionally and anatomically: the activation of the left and right anterior VLPFC is specifically associated with *similarity detection*, while the activation of the left DLPFC is associated with *abstraction*. This result supports our working hypothesis based on clinical observations of differential categorization deficits in patients with PFC lesions. The findings of this study also shed new light on the role of the anterior VLPFC in *similarity detection*, a structure-function relationship that has not been clearly established until now. Additionally, this study shows a relative left-right dissociation according to the type of similarity to be detected (physical or conceptual).

### 1. The brain network involved in Similarity detection

Using the experimental paradigm above, we were able to show that the anterior VLPFC is involved when subjects have to detect the most similar object, rather than when they have to find the most different one. It is unlikely that *difficulty* is a critical parameter for the activation of the anterior VLPFC in this experimental context. Indeed, shorter reaction times are observed in *same* tasks (*same shape* or *same category*) than in *different* tasks (*different shape* or *different category*). These data indicate that it may be more natural and easier to detect similarities than differences.

Although these results support the particular involvement of the anterior VLPFC in *similarity* detection, one may hypothetically argue that in *different* conditions, detecting similar objects is a pre-required process in order to detect difference. This hypothesis is supported by the slightly longer reaction times for *different* than for *same* conditions. If true, this hypothesis implies that subjects have to judge which object is the most similar to select the alternative one. That is to say that *different* conditions involve two processes (*similarity* then *difference*) while *same* conditions only involve *similarity detection*. Opposite to this hypothesis, the absence of detection of prefrontal activation in the *different>same* contrast (Table 1) combined to the strong anterior VLPFC activation in the opposite contrast (*same*>*different*) give strength to the conclusion that extra cognitive control is necessary for *similarity detection*, as compared to difference detection. In addition, longer reaction times in the *different* conditions may also be explained by the longer time required to find out that two objects are different as compared as finding out similarities.

Our results suggest that the anterior VLPFC plays a key role in *similarity detection*, a function that has not previously been identified. The VLPFC has been shown to be associated with various cognitive functions, such as set-shifting, rule learning and rule use, retrieval and selection of semantic knowledge and of relevant information among memory traces, on-line maintenance during working memory tasks and analogical reasoning [4], [13]–[26]. At first sight, there seems to be no obvious relationship between the above functions and *similarity detection*. How do the results of our study fit in with these previous findings? First, a simple explanation is that the neural basis of *similarity detection* has never been studied because the hypothesis that specific areas are involved in such processing has not as yet been formulated. The clinical observation of some patients with prefrontal damage (see the attached video), showing the difficulty of detecting physical or abstract similarities between objects in categorization tasks, nevertheless suggests that *similarity detection* occupies a functionally and anatomically discrete region within the PFC. Second, the anatomical and functional heterogeneity of the VLPFC can account for the vast spectrum of functions associated with it. We have shown in the present study that activation related to *similarity detection* is associated with the anterior VLPFC, while more posterior VLPFC subareas are included in a network involved in *abstraction*. Third, the present data pinpoint a fundamental function of the anterior VLPFC that could unify several of the other functions or processes associated with this structure. Rule elaboration, retrieval and selection of semantic knowledge, and analogical reasoning all require that similarities between physical objects or abstract items present in the environment or stored in memory be identified or retrieved, although it is not possible from our results to definitely prove that the above functions hierarchically depend on *similarity detection*. The manner in which the anterior VLPFC is involved in *similarity detection* also remains speculative. Indeed, *similarity detection* may depend on more elementary sub-processes such as maintaining the intention to search for identity [27], building mental representations of abstract and/or concrete features for all items, selecting relevant representations and inhibiting non-relevant ones [15], or using these representations to match items. Further studies are needed to verify whether the mechanisms proposed here for similarity detection depend on the anterior VLPFC.

In ROI analyses, we observe a left/right asymmetry in the anterior VLPFC, with the right anterior VLPFC being specifically engaged in perceptual similarities while the left anterior VLPFC is engaged in both perceptual and abstract relationships. These results are consistent with previous studies. Milton et al. [28] have shown the involvement of the right ventral PFC in perceptual similarity sorting. The left rostral PFC has been associated with analogical reasoning, a process that requires the detection of conceptual similarities between items [29], [4], [30]. Moreover, Bunge et al. [31] have suggested a lateralization of relational integration, a process that requires the detection of similarities: the right rostral and lateral PFC would play a more active role in processing visuospatial relationships than the left, whereas the analogous region on the left would play a more active role in processing verbal or semantic relationships. The activated foci found in the Bunge et al. [31] study are anatomically close to the ones seen in our study, although their activated regions are slightly more anterior and less ventral than ours. Taken as a whole, the present data combined with previous studies suggest that the right anterior VLPFC contributes to concrete thinking and particularly to the process of detecting physical similarities between items, whereas the left anterior VLPFC tends to be more involved in finding conceptual relationships between items.

### 2. The brain network involved in abstraction

Our data also show the involvement of the left dorsolateral PFC in *abstraction*, i.e. when subjects have to indicate whether items can be grouped or separated according to taxonomic or thematic categories (*same* and *different category* conditions) as opposed to when subjects are asked to compare items according to their visual shape, regardless of their category (*same* and *different shape* conditions). This result is in accordance with a recent study showing a decrease in the volume of the left PFC (as assessed by MRI volume-based morphometry) in patients presenting with conceptualization deficits in the context of neurodegenerative dementias [32]. Interestingly, recent works suggest a rostro-caudal model organization of the PFC for abstraction, the most anterior regions being recruited for more abstract tasks [33], [34]. In accordance with this model, thematic and taxonomic categories used in our study are sufficient to activate a relatively caudal portion of the left DLPFC. However, the level of abstraction reached in the present study is far from the type of abstract processing required to activate the rostrolateral PFC [33], [34]. Several interrelated explanations can be proposed for a role of the left dorsolateral PFC in *abstraction*: First, it is likely that the left PFC activation observed in our study is at least partly related to language production, as has already been shown [32], [35]–[37]. Indeed, the categorization task used in our study requires the activation of taxonomic or thematic categories that are generally verbally coded. Second, abstract categorization (i.e. the organization of knowledge according to abstract ideas that go beyond the physical features of objects) relies on other functions such as semantic judgment or strategies for the selection of appropriate conceptual knowledge [32], [37]. These functions have been associated with the left PFC in several imaging studies [38]–[48]. Third, as the lateral PFC is also critical for all the so-called executive functions and cognitive control [49], one could question the functional relationship between *abstraction* and executive functions. For instance, because of the large number of categories and the different levels of classification used in our study, it is likely that finding an abstract category was based on inductive reasoning and the generation of hypotheses, two important executive processes involving the lateral PFC [50]–[53].

### 3. Control of experimental conditions

The reliability of these findings results from the control of several critical task parameters. First, these results were obtained by the use of a factorial task design, orthogonally crossing *similarity detection* and *abstraction*. This task design allowed us to study the two dimensions separately and to look for interactions between them. Second, several different categories of items (240) and levels of abstract classification (taxonomic, thematic, ordinal, supra-ordinal, spatial-temporal, causal…) were used in order to ensure that any changes in brain signals were not related to a specific category or group of items. Third, in a preliminary psychometric study carried out prior to the fMRI study, we eliminated any item or trial that was perceived by the participants as ambiguous in terms of shape or semantic link. Fourth, motor responses were equally distributed between the left and right sides (see Supplementary material). Fifth, matching and non-matching trials were equally distributed among the conditions to avoid the influence of shape on a category decision and vice versa (see Supplementary material and Fig. 1). Sixth, four different versions of the paradigm, each used for five participants (see Supplementary material and Table S1), were created such that each stimulus was seen only once by a given subject, but was used an equal number of times in every condition. This was carried out to ensure that any changes in brain signals were not related to differences in the stimuli used under different conditions. Finally, the order of trials and conditions was randomized for each participant.

### 4. Are similarity detection and abstraction independent of each other?

We have shown here that the two processes- *abstraction* and *similarity detection* - might be functionally and anatomically discrete, consistent with our clinical observations in frontal lesion patients. This suggests that each of these processes could be altered independently of the other. However, an important issue that should be addressed in future studies is to determine whether the anatomical-functional dissociation between *similarity detection* and *abstraction* is complete or incomplete. In other words, is it possible to use abstract thinking without searching for similarities between items or to detect similarities without abstract thinking? Our data, showing the anatomical proximity between the area activated during *similarity detection*, the left anterior VLPFC, and the area involved in both *similarity detection* and *abstraction*, the left mid-VLPFC, indicate at least an anatomical-functional continuum between the areas involved in the two types of processes. Accordingly, one could hypothesize a dissociation of deficits depending on the location of the lesion (right/left rostral ventrolateral or left dorsolateral and prefrontal regions).

The identification of the discrete PFC areas involved in *similarity detection* and *abstraction* should provide a stronger basis for the conduction of clinical-anatomical correlation studies in patients with frontal lobe lesions.

## Supporting Information

Text S1 **Conception of stimuli.**
(DOCX)

Text S2 **Control of the critical parameters of the experimental procedure.**
(DOCX)

Video S1 **This video shows 2 patients who have a frontal lobe syndrome and who exhibit two types of abnormal behaviour when asked to place a banana and an orange in a single category: The first patient places them at a concrete level (e.g., “They are eatable”), while the second looks for differences between these objects (e.g., “they don't look alike etc.”).**
(MOV)

Figure S1 **Accuracy (fMRI study).** Histograms represent means +/− standard errors of the mean. *: *p*<.05; **: *p*<.01; ***: *p*<.001. **a.** Comparison of mean error rate for *category* (Same Category and Different Category) and *shape* (same shape and different shape) conditions. Paired *t*-tests were used for comparisons. Diagrams show that there were significantly more errors under *category* (mean ± SD: 5.5±2.7%) than under *shape* (mean ± SD: 4.1±2.3%) conditions (T[19] = 3.43, *p*<0.001). **b.** Mean error rate for *same* and *different* conditions. Paired *t*-tests were used for comparisons, and showed no significant difference between the conditions (T[19] = 0.84, *p* = 0.4). **c.** Comparison of the mean error rate across the four conditions. ANOVA and Tukey's post hoc analyses were used for comparisons. SSh: Same Shape, DSh: Different Shape, SCat: Same Category, DCat: Different Category. ANOVA revealed that the effect of “condition” on the error rate was significant (F[3,19] = 4.243; *p*<0.009). Post hoc analyses confirmed a significant difference between the different shape (mean ± SD: 3.78±1.83%) and same category or different category conditions (mean ± SD: 5.48± 2.6%, in both same and different category).
(DOCX)

Figure S2 **Reaction times and percentage of errors over the eight sessions (fMRI study).** Repeated measures ANOVA were performed to compare error rates across the eight sessions. a. Histograms represent mean reaction times +/− standard errors of the mean during the eight sessions. Repeated measures ANOVA revealed no difference in RT during the experiment. b. Histograms represent the mean error rate +/− standard error of the mean during the eight sessions. ANOVA and post hoc analysis revealed that there were more errors during the first session as compared to session 5 and session 8.
(DOCX)

Table S1 **Distribution of stimuli in four versions of the paradigm.**
(DOCX)

Table S2 **Results of the main contrasts of interest with RT as covariate.**
(DOCX)

## References

[1] Ashby FG, Maddox WT (2011). Human category learning 2.0.. Ann N Y Acad Sci.

[2] Smith EE, Grossman M (2008). Multiple systems of category learning.. Neurosci Biobehav Rev.

[3] Dubois B, Slachevsky A, Litvan I, Pillon B (2000). The FAB: a Frontal Assessment Battery at bedside.. Neurology.

[4] Bunge SA, Wendelken C, Badre D, Wagner AD (2005). Analogical reasoning and prefrontal cortex: evidence for separable retrieval and integration mechanisms.. Cereb Cortex.

[5] Pilgrim LK, Fadili J, Fletcher P, Tyler LK (2002). Overcoming confounds of stimulus blocking: an event-related fMRI design of semantic processing.. Neuroimage.

[6] Adams RB, Janata P (2002). A comparison of neural circuits underlying auditory and visual object categorization.. Neuroimage.

[7] Devlin JT, Russell RP, Davis MH, Price CJ, Moss HE (2002). Is there an anatomical basis for category-specificity? Semantic memory studies in PET and fMRI.. Neuropsychologia.

[8] Tyler LK, Russell R, Fadili J, Moss HE (2001). The neural representation of nouns and verbs: PET studies.. Brain.

[9] Vogels R, Sary G, Dupont P, Orban GA (2002). Human brain regions involved in visual categorization.. Neuroimage.

[10] Reber PJ, Wong EC, Buxton RB (2002). Comparing the brain areas supporting nondeclarative categorization and recognition memory.. Brain Res Cogn Brain Res.

[11] Friston KJ, Holmes A, Poline JB, Price CJ, Frith CD (1996). Detecting activations in PET and fMRI: levels of inference and power.. Neuroimage.

[12] Brett M, Anton J-L, Valabregue R, Pioline J-B (2002). Region of interest analysis using an SPM toolbox [abstract] Presented at the 8th Internation Conference on Functional Mapping of the Human Brain,..

[13] Petrides M, Alivisatos B, Evans AC (1995). Functional activation of the human ventrolateral frontal cortex during mnemonic retrieval of verbal information.. Proc Natl Acad Sci USA.

[14] Dias R, Robbins TW, Roberts AC (1997). Dissociable forms of inhibitory control within prefrontal cortex with an analog of the Wisconsin Card Sort Test: restriction to novel situations and independence from “on-line” processing.. J Neurosci.

[15] Thompson-Schill SL, D'Esposito M, Aguirre GK, Farah MJ (1997). Role of left inferior prefrontal cortex in retrieval of semantic knowledge: a reevaluation.. Proc Natl Acad Sci U S A.

[16] Martin A, Chao LL (2001). Semantic memory and the brain: structure and processes.. Curr Opin Neurobiol.

[17] Nakahara K, Hayashi T, Konishi S, Miyashita Y (2002). Functional MRI of macaque monkeys performing a cognitive set-shifting task.. Science.

[18] Bunge SA, Kahn I, Wallis JD, Miller EK, Wagner AD (2003). Neural circuits subserving the retrieval and maintenance of abstract rules.. J Neurophysiol.

[19] Bunge SA (2004). How we use rules to select actions: a review of evidence from cognitive neuroscience.. Cogn Affect Behav Neurosci.

[20] Sakai K, Passingham RE (2003). Prefrontal interactions reflect future task operations.. Nat Neurosci.

[21] Badre D, Poldrack RA, Paré-Blagoev EJ, Insler RZ, Wagner AD (2005). Dissociable controlled retrieval and generalized selection mechanisms in ventrolateral prefrontal cortex.. Neuron.

[22] Geake JG, Hansen PC (2005). Neural correlates of intelligence as revealed by fMRI of fluid analogies.. Neuroimage.

[23] Badre D, Wagner AD (2007). Left ventrolateral prefrontal cortex and the cognitive control of memory.. Neuropsychologia.

[24] Kostopoulos P, Petrides M (2008). Left mid-ventrolateral prefrontal cortex: underlying principles of function.. Eur J Neurosci.

[25] Qiu J, Li H, Chen A, Zhang Q (2008). The neural basis of analogical reasoning: an event-related potential study.. Neuropsychologia.

[26] Wendelken C, Nakhabenko D, Donohue SE, Carter CS, Bunge SA (2008). “Brain is to thought as stomach is to…?”: investigating the role of rostrolateral prefrontal cortex in relational reasoning.. J Cogn Neurosci.

[27] Volle E, Gilbert SJ, Benoit RG, Burgess PW (2010). Specialization of the rostral prefrontal cortex for distinct analogy processes.. Cereb Cortex.

[28] Milton F, Wills AJ, Hodgson TL (2009). The neural basis of overall similarity and single-dimension sorting.. Neuroimage.

[29] Green AE, Fugelsang JA, Kraemer DJM, Shamosh NA, Dunbar KN (2006). Frontopolar cortex mediates abstract integration in analogy.. Brain Res.

[30] Boroojerdi B, Phipps M, Kopylev L, Wharton CM, Cohen LG (2001). Enhancing analogic reasoning with rTMS over the left prefrontal cortex.. Neurology.

[31] Bunge SA, Helskog EH, Wendelken C (2009). Left, but not right, rostrolateral prefrontal cortex meets a stringent test of the relational integration hypothesis.. Neuroimage.

[32] Fine EM, Delis DC, Dean D, Beckman V, Miller BL (2009). Left frontal lobe contributions to concept formation: a quantitative MRI study of performance on the Delis-Kaplan Executive Function System Sorting Test.. J Clin Exp Neuropsychol.

[33] Christoff K, Keramatian K, Gordon AM, Smith R, Mädler B (2009). Prefrontal organization of cognitive control according to levels of abstraction.. Brain Res.

[34] Badre D (2008). Cognitive control, hierarchy, and the rostro-caudal organization of the frontal lobes.. Trends Cogn Sci (Regul Ed).

[35] Broca P (1861). Remarques sur le siège de la faculté du language articulé suivies d'une observation d'aphémie (perte de la parole).. Bulletin de la Société Anatomique.

[36] Geschwind N (1970). The organization of language and the brain.. Science.

[37] Delis DC, Kaplan E, Kramer JH (2001). D-KEFS..

[38] Démonet JF, Chollet F, Ramsay S, Cardebat D, Nespoulous JL (1992). The anatomy of phonological and semantic processing in normal subjects.. Brain.

[39] Demb JB, Desmond JE, Wagner AD, Vaidya CJ, Glover GH (1995). Semantic encoding and retrieval in the left inferior prefrontal cortex: a functional MRI study of task difficulty and process specificity.. J Neurosci.

[40] Spitzer M, Kwong KK, Kennedy W, Rosen BR, Belliveau JW (1995). Category-specific brain activation in fMRI during picture naming.. Neuroreport.

[41] Vandenberghe R, Price C, Wise R, Josephs O, Frackowiak RS (1996). Functional anatomy of a common semantic system for words and pictures.. Nature.

[42] Cappa SF, Perani D, Schnur T, Tettamanti M, Fazio F (1998). The effects of semantic category and knowledge type on lexical-semantic access: a PET study.. Neuroimage.

[43] Dalla Barba G, Parlato V, Jobert A, Samson Y, Pappata S (1998). Cortical networks implicated in semantic and episodic memory: common or unique?. Cortex.

[44] Perani D, Schnur T, Tettamanti M, Gorno-Tempini M, Cappa SF (1999). Word and picture matching: a PET study of semantic category effects.. Neuropsychologia.

[45] Poldrack RA, Wagner AD, Prull MW, Desmond JE, Glover GH (1999). Functional specialization for semantic and phonological processing in the left inferior prefrontal cortex.. Neuroimage.

[46] Friederici AD, Opitz B, von Cramon DY (2000). Segregating semantic and syntactic aspects of processing in the human brain: an fMRI investigation of different word types.. Cereb Cortex.

[47] Gerlach C, Law I, Gade A, Paulson OB (2000). Categorization and category effects in normal object recognition: a PET study.. Neuropsychologia.

[48] Wood JN, Romero SG, Makale M, Grafman J (2003). Category-specific representations of social and nonsocial knowledge in the human prefrontal cortex.. J Cogn Neurosci.

[49] Duncan J, Owen AM (2000). Common regions of the human frontal lobe recruited by diverse cognitive demands.. Trends Neurosci.

[50] Goel V, Dolan RJ (2000). Anatomical segregation of component processes in an inductive inference task.. J Cogn Neurosci.

[51] Goel V, Dolan RJ (2004). Differential involvement of left prefrontal cortex in inductive and deductive reasoning.. Cognition.

[52] Reverberi C, Lavaroni A, Gigli GL, Skrap M, Shallice T (2005). Specific impairments of rule induction in different frontal lobe subgroups.. Neuropsychologia.

[53] Reverberi C, D'Agostini S, Skrap M, Shallice T (2005). Generation and recognition of abstract rules in different frontal lobe subgroups.. Neuropsychologia.

