# Supplementary material for: Similar or Different? The Role of the Ventrolateral Prefrontal Cortex in Similarity Detection
Source: PLoS One. 2012 Mar 30;7(3):e34164. doi: 10.1371/journal.pone.0034164 (PMC3316621; doi:10.1371/journal.pone.0034164)
Supplement: Text S1 — Conception of stimuli. (DOCX) [file pone.0034164.s001.docx]

**Text S1. Conception of stimuli**

We built a total of 600 slides (“stimuli”): 576 slides for the experiment and 24 for the training session. Line drawings were collected from normalized batteries of black-and-white line drawings [1], [2] neuropsychological tests such as the British Picture Vocabulary Scale [3] and free-download coloring websites ([www.coloriage.tfou.fr](http://www.coloriage.tfou.fr), [www.hugolescargot.com](http://www.hugolescargot.com), [www.mescoloriages.com](http://www.mescoloriages.com), [www.coloriage.info](http://www.coloriage.info), [www.jedessine.com](http://www.jedessine.com), [www.jolicoloriage.free](http://www.jolicoloriage.free), [www.coloriage.gulli.fr](http://www.coloriage.gulli.fr)). The aspect of the drawings was standardized with the software Adobe Photoshop CS ([www.adobe.com](http://www.adobe.com)). To ensure that conceptual and shape relationships were real and strong, a pre-test was conducted. Twelve volunteers who did not participate in the fMRI experiment (aged 20 to 27 years, French native speakers, right-handed, with normal visual acuity) were asked to perform the experimental paradigm as described in the methods. All subjects were enrolled at the University of Paris VI. There was a debriefing after they had performed the tasks, in which they were questioned about the difficulty, timing and length of the session and task conditions. They were asked to comment on each trial in which they gave the wrong response, and any trial for which they reported an ambiguity in the shape or the semantic link was removed before the next pre-test subject. We ended the pre-tests when the error rate was below 5% for four consecutive participants.

**References**

1. Sirois M, Kremin H, Cohen H (2006) Picture-naming norms for Canadian French: name agreement, familiarity, visual complexity, and age of acquisition. Behav Res Methods 38: 300–306.

2. Snodgrass JG, Vanderwart M (1980) A standardized set of 260 pictures: norms for name agreement, image agreement, familiarity, and visual complexity. J Exp Psychol Hum Learn 6: 174–215.

3. Dunn L, Dunn L, Whetton C, Bruley J (1997) Britishu Picture Vocabulary Scale, Second Edition (The). NFER-Nelson. p.
