# Supplementary material for: Similar or Different? The Role of the Ventrolateral Prefrontal Cortex in Similarity Detection
Source: PLoS One. 2012 Mar 30;7(3):e34164. doi: 10.1371/journal.pone.0034164 (PMC3316621; doi:10.1371/journal.pone.0034164)
Supplement: Text S2 — Control of the critical parameters of the experimental procedure. (DOCX) [file pone.0034164.s002.docx]

**Text S2. Control of the critical parameters of the experimental procedure**

To balance motor activation between the left and the right sides, correct responses were equally distributed between the bottom-left and bottom-right drawings for every condition. To ensure that there was no influence of shape on a category decision and vice versa, matching and non-matching slides were equally distributed for every condition. In order to use every stimulus in every condition and to ensure that each participant saw a given stimulus only once, we divided the stimuli into four sets of 144 slides, and created four versions of the paradigm (cf. Table S1). In each set, we put 36 non-matching slides with a similar-shape drawing on the left (cf. Fig 1A) together with 36 non-matching slides with a similar-shape drawing on the right (cf. Fig 1B), 36 matching slides with a similar-shape drawing on the left (cf. Fig 1 C) and 36 matching slides with a similar-shape drawing on the right (cf Fig 1D). This distribution ensured symmetry between tasks. In each version, stimuli from a specific set were used for a specific condition as represented in the supplementary table. Each version was used 5 times (i.e. for 5 participants). Furthermore, to control for bias due to the order of presentation of the slides and conditions, we randomized these for each participant.
