# Supplementary material for: Similar or Different? The Role of the Ventrolateral Prefrontal Cortex in Similarity Detection
Source: PLoS One. 2012 Mar 30;7(3):e34164. doi: 10.1371/journal.pone.0034164 (PMC3316621; doi:10.1371/journal.pone.0034164)
Supplement: Figure S1 — Accuracy (fMRI study). Histograms represent means +/− standard errors of the mean. *: p<.05; **: p<.01; ***: p<.001. a. Comparison of mean error rate for category (Same Category and Different Category) and shape (same shape and different shape) conditions. Paired t-tests were used for comparisons. Diagrams show that there were significantly more errors under category (mean ± SD: 5.5±2.7%) than under shape (mean ± SD: 4.1±2.3%) conditions (T[19] = 3.43, p<0.001). b. Mean error rate for same and different conditions. Paired t-tests were used for comparisons, and showed no significant difference between the conditions (T[19] = 0.84, p = 0.4). c. Comparison of the mean error rate across the four conditions. ANOVA and Tukey's post hoc analyses were used for comparisons. SSh: Same Shape, DSh: Different Shape, SCat: Same Category, DCat: Different Category. ANOVA revealed that the effect of “condition” on the error rate was significant (F[3,19] = 4.243; p<0.009). Post hoc analyses confirmed a significant difference between the different shape (mean ± SD: 3.78±1.83%) and same category or different category conditions (mean ± SD: 5.48± 2.6%, in both same and different category). (DOCX) [file pone.0034164.s004.docx]

**Figure S1. Accuracy (fMRI study).**

**
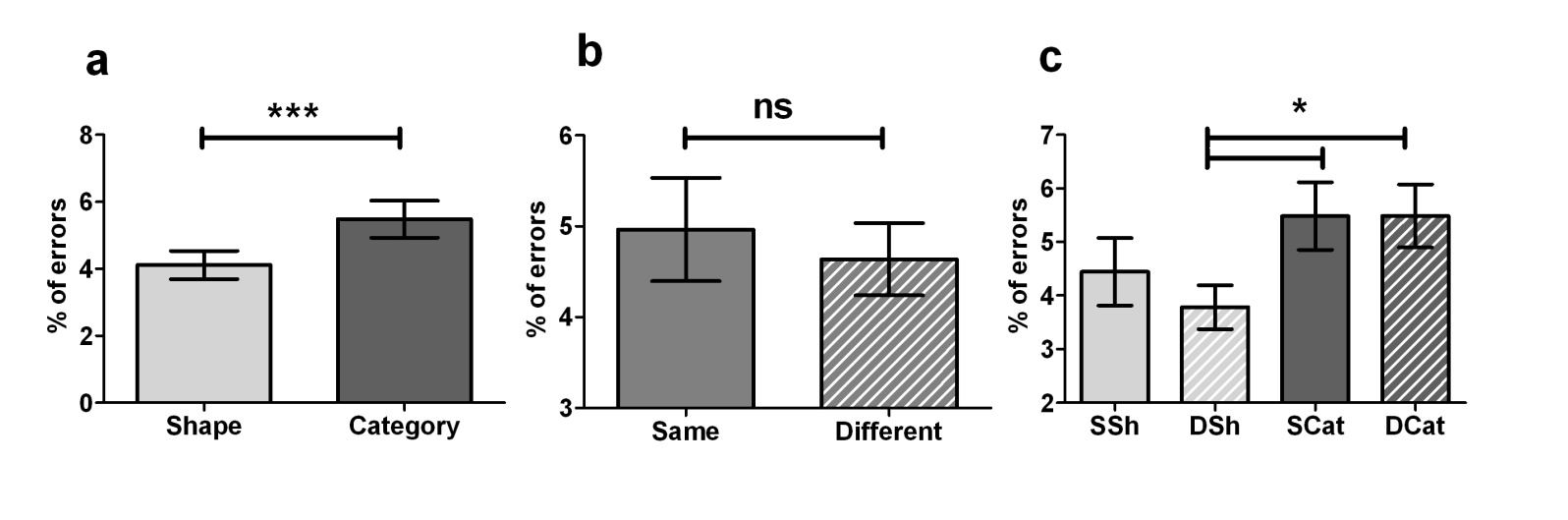
**

Histograms represent means +/- standard errors of the mean. *: *p* < .05; **: *p* < .01; ***: *p* < .001. **a.** Comparison of mean error rate for *category* (Same Category and Different Category) and *shape* (same shape and different shape) conditions. Paired *t*-tests were used for comparisons. Diagrams show that there were significantly more errors under *category* (mean ± SD: 5,5 ± 2,7 %) than under *shape* (mean ± SD: 4,1 ± 2,3 %) conditions (T[19] = 3,43, *p* < 0,001)**.**. **b.** Mean error rate for *same* and *different* conditions. Paired *t*-tests were used for comparisons, and showed no significant difference between the conditions (T[19] = 0,84, *p* = 0,4). **c.** Comparison of the mean error rate across the four conditions. ANOVA and Tukey's post hoc analyses were used for comparisons. SSh: Same Shape, DSh: Different Shape, SCat: Same Category, DCat: Different Category. ANOVA revealed that the effect of “condition” on the error rate was significant (F[3,19] = 4,243; *p* < 0.009). Post hoc analyses confirmed a significant difference between the different shape (mean ± SD: 3.78 ±1.83 %) and same category or different category conditions (mean ± SD: 5.48 ± 2.6 %, in both same and different category).
