# Supplementary material for: Similar or Different? The Role of the Ventrolateral Prefrontal Cortex in Similarity Detection
Source: PLoS One. 2012 Mar 30;7(3):e34164. doi: 10.1371/journal.pone.0034164 (PMC3316621; doi:10.1371/journal.pone.0034164)
Supplement: Figure S2 — Reaction times and percentage of errors over the eight sessions (fMRI study). Repeated measures ANOVA were performed to compare error rates across the eight sessions. a. Histograms represent mean reaction times +/− standard errors of the mean during the eight sessions. Repeated measures ANOVA revealed no difference in RT during the experiment. b. Histograms represent the mean error rate +/− standard error of the mean during the eight sessions. ANOVA and post hoc analysis revealed that there were more errors during the first session as compared to session 5 and session 8. (DOCX) [file pone.0034164.s005.docx]

**Figure S2. Reaction times and percentage of errors over the eight sessions (fMRI study).**

**
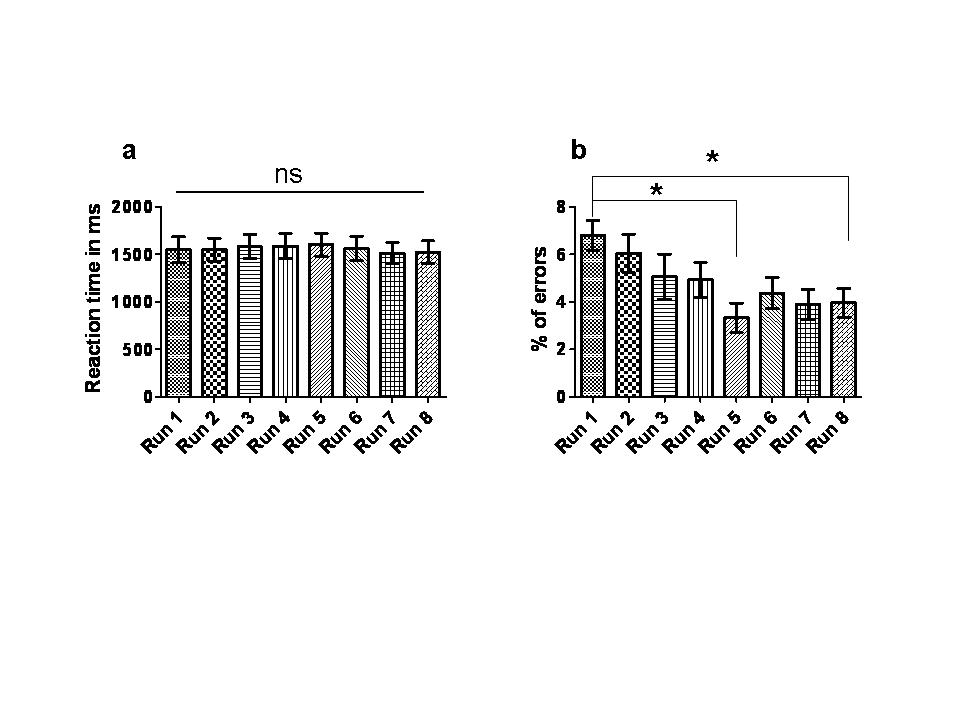
**

Repeated measures ANOVA were performed to compare error rates across the eight sessions. a. Histograms represent mean reaction times +/- standard errors of the mean during the eight sessions. Repeated measures ANOVA revealed no difference in RT during the experiment. b. Histograms represent the mean error rate +/- standard error of the mean during the eight sessions. ANOVA and post hoc analysis revealed that there were more errors during the first session as compared to session 5 and session 8.
