# Supplementary material for: Similar or Different? The Role of the Ventrolateral Prefrontal Cortex in Similarity Detection
Source: PLoS One. 2012 Mar 30;7(3):e34164. doi: 10.1371/journal.pone.0034164 (PMC3316621; doi:10.1371/journal.pone.0034164)
Supplement: Table S1 — Distribution of stimuli in four versions of the paradigm. (DOCX) [file pone.0034164.s006.docx]

**Table S1. Distribution of stimuli in four versions of the paradigm.**

SSh: Same Shape, DSh: Different Shape, SCat: Same Category, DCat: Different Category The table shows how stimuli were distributed among four sets. Each set contained an equal number of matching and non-matching stimuli, as well as an equal number of correct answers on the right and on the left, as explained above. Each set was used five times for each condition.

| **Set of stimuli** | **Version 1** | **Version 2** | **Version 3** | **Version 4** |
| --- | --- | --- | --- | --- |
| **Set 1**  **(144 stimuli)** | Used in SSh condition | Used in SCat condition | Used in DSh condition | Used in DCat condition |
| **Set 2**  **(144 stimuli)** | Used in SCat Condition | Used in DSh condition | Used in DCat condition | Used in SSh Condition |
| **Set 3**  **(144 stimuli)** | Used in DSh Condition | Used in DCat condition | Used in SSh Condition | Used in SCat condition |
| **Set 4**  **(144 stimuli)** | Used in DCat Condition | Used in SSh condition | Used in SCat condition | Used in DSh condition |
