# Supplementary material for: Similar or Different? The Role of the Ventrolateral Prefrontal Cortex in Similarity Detection
Source: PLoS One. 2012 Mar 30;7(3):e34164. doi: 10.1371/journal.pone.0034164 (PMC3316621; doi:10.1371/journal.pone.0034164)
Supplement: Table S2 — Results of the main contrasts of interest with RT as covariate. (DOCX) [file pone.0034164.s007.docx]

**Table S2. Results of the main contrasts of interest with RT as covariate**

The table shows all clusters surviving a FWE correction when RT was added to the model as a variate of non-interest. (*p* < .05) *: *p* < .05; **: *p* < .01; ***: *p* < .001.

| ***Contrast*** | ***Region*** | ***Side*** | ***BA*** | ***MNI coordinate*** | ***z*** |
| --- | --- | --- | --- | --- | --- |
| **Category > Shape** | |  |  |  |  |
|  | middle/superior frontal gyrus | L | 8/9 | -30 26 56 | 4.78*** |
|  | inferior frontal gyrus | L | 44 | -46 16 30  -52 22 38 | 4.50*** |
|  | fusiform gyrus/cerebellum | R | 37/18/20 | 34 -46 -22 | 4.66*** |
|  | fusiform gyrus/cerebellum | L | 37/19 | -28 -64 -18 | 4,22*** |
|  | fusiform gyrus/cingulate gyrus | L | 37/30 | -26 -36 -20 | 4.37*** |
|  | angular gyrus/middle occipital gyrus/inferior parietal cortex | L | 7/19/39 | -32 -70 38 | 4.55*** |
|  | middle temporal gyrus | L | 21/22 | -54 -46 -2 | 4.16** |
|  | middle temporal gyrus/angular gyrus | R | 39 | 48 -64 26 | 3,90** |
|  | inferior/middle occipital gyrus | R | 18/19 | 34 -94 -10 | 4.96*** |
|  | inferior/middle occipital gyrus | L | 18/19 | -28 -96 10 | 4.68** |
| **Shape > Category** | |  |  |  |  |
|  | supramarginal gyrus/ inferior and superior parietal cortex | L | 2/7/40 | -60 -28 42 | 5.76*** |
|  | Superior parietal cortex | R | 7 | 18 -50 54 | 4.17** |
|  | supramarginal gyrus/ inferior parietal cortex | R | 2/40 | 56 -22 40 | 5.90*** |
| **Same > Different** | |  |  |  |  |
|  | inferior frontal orbital cortex/inferior frontal gyrus | R | 45/46/47/11 | 46 48 0 | 5.12** |
|  | inferior frontal orbital cortex | L | 47 | -44 46 -12 | 4.92* |
| **Different > Same** | |  |  |  |  |
|  | superior parietal cortex/precuneus | R | 5/7 | 12 -64 60 | 5.36*** |
